# DGAT1 regulates keratinocyte proliferation through the modulation of retinoid homeostasis

**DOI:** 10.1007/s13105-026-01196-w

**Published:** 2026-06-10

**Authors:** Kamila Kwiecień, Natalia Bryniarska-Kubiak, Agnieszka Morytko, Alicja Uchańska, Maciej Pastuszczak, Marcin Migaczewski, Joanna Cichy, Patrycja Kwiecińska

**Affiliations:** 1 Department of Immunology, Faculty of Biochemistry, Biophysics and Biotechnology, Jagiellonian University, Kraków, Poland; 2 Laboratory of Mucosal Immunology, Translational Research Center for Gastrointestinal Disorders (TARGID), Department of Chronic Diseases and Metabolism (CHROMETA), KU Leuven, Leuven, Belgium; 3 Laboratory of Stem Cell Biology, Faculty of Biochemistry, Biophysics and Biotechnology, Jagiellonian University, Kraków, Poland; 4 Selvita S.A, Kraków, Poland; 5 Clinical Department of Dermatology, Medical University of Silesia, Zabrze, Poland; 6 Department of General Surgery, Jagiellonian University Medical College, Kraków, Poland

**Keywords:** DGAT1, Keratinocytes, Epidermis, Psoriasis, Retinoids

## Abstract

Multiple mechanisms contribute to skin barrier integrity, and emerging evidence suggests that acyl-CoA:diacylglycerol acyltransferase 1 (DGAT1) plays a key role in its regulation through both lipid and retinoid metabolism. Although DGAT1 deficiency leads to cutaneous abnormalities, the direct role of DGAT1 in keratinocyte biology remains unclear. Here, we show that DGAT1 deficiency drives extensive keratinocyte hyperproliferation and barrier dysfunction under homeostatic conditions. Transcriptomic profiling revealed upregulation of genes associated with proliferation, inflammation, and retinoic acid (RA) signaling, consistent with increased proliferation observed *in vitro* and *in vivo*. Unexpectedly, while DGAT1 expression was elevated in human psoriatic lesions and in a murine psoriasis model, DGAT1 loss reduced keratinocyte proliferation in this inflammatory condition. Further analyses identified dysregulated RA signaling as a central mediator of these effects. DGAT1-deficient keratinocytes exhibited elevated RA-target gene expression and increased expression of RA-binding proteins, indicating enhanced RA bioavailability. Our findings establish DGAT1 as a context-dependent regulator of keratinocyte proliferation, promoting growth in homeostasis while restraining it in psoriasis. By linking DGAT1 activity with RA signaling, this work uncovers a previously unrecognized pathway contributing to epidermal physiology and inflammatory skin disease.

**Supplementary Information:**

The online version contains supplementary material available at 10.1007/s13105-026-01196-w.

## Introduction

The skin, the largest organ of the human body, provides a physical and biochemical barrier that protects against environmental insults, pathogens, and excessive water loss [1]. This function is primarily mediated by keratinocytes, which form stratified layers during differentiation. The outermost layer, the stratum corneum, consists of corneocytes embedded in a lipid-rich extracellular matrix, while keratinocytes in the stratum granulosum are connected by tight junctions. Together, these structures restrict transepidermal water loss (TEWL) and regulate molecular passage across the skin [2]. Barrier disruption is a key pathogenic feature of several skin disorders, including atopic dermatitis, psoriasis, ichthyosis, and contact dermatitis [3].

Multiple mechanisms contribute to skin barrier integrity, and emerging evidence suggests that acyl-CoA: diacylglycerol acyltransferase 1 (DGAT1) plays a role in its regulation. DGAT1 is one of two enzymes catalyzing the final step of triglyceride (TG) synthesis in mammals. In addition to its metabolic functions, DGAT1 deficiency results in cutaneous abnormalities. *Dgat1* knockout mice exhibit altered fur lipid composition, sebaceous gland atrophy, alopecia, and increased TEWL [4–6], highlighting its importance in maintaining barrier integrity. These phenotypes are attributed to impaired retinoid homeostasis, since DGAT1 also functions as an acyl-CoA: retinol acyltransferase (ARAT), converting retinol into retinyl esters [7]. Its loss is associated with elevated retinoic acid (RA) levels in the skin, leading to epidermal hyperplasia in response to dietary or topical retinol supplementation, without systemic retinoid alterations.

Retinoids (vitamin A derivatives) have long been recognized as central regulators of skin physiology, with both deficiency and excess producing pathological effects. By the 1930 s, vitamin A deficiency was identified as a cause of skin hyperkeratosis [8]. When applied therapeutically at appropriate doses, RA effectively treats hyperproliferative and inflammatory skin conditions such as acne, psoriasis, ichthyosis, and skin cancers through its effects on keratinocyte differentiation and immune modulation [9–13]. Within keratinocytes, RA serves as a potent signaling molecule that regulates gene expression via retinoic acid receptors (RARs) and retinoid X receptors (RXRs), thereby controlling key processes including proliferation, differentiation, lipid metabolism, and apoptosis [14, 15]. The activity of RA is tightly regulated by enzymes and binding proteins that modulate its intracellular bioavailability [16].

Despite these insights, the direct impact of DGAT1 on keratinocytes, the primary cell type responsible for the epidermal barrier, remains poorly understood. Here, we show that DGAT1 loss exerts a dual effect on murine keratinocytes: it promotes hyperproliferation under physiological conditions but reduces proliferation in psoriatic skin. Given the established role of DGAT1 in retinoid metabolism, we propose that these effects are mediated by elevated retinoic acid levels resulting from DGAT1 deficiency.

## Materials and methods

### Human skin biopsies

All human studies were conducted in accordance with the guidelines of the Jagiellonian University Institutional Bioethics Committee (protocol no. 1072.6120.30.2020) and adhered to the Declaration of Helsinki. Written informed consent was obtained from all participants prior to sample collection. Six-millimeter punch biopsies were taken under local anesthesia from lesional skin of patients with psoriasis (*n* = 8; female: male ratio 3:5; mean age 44 ± 16 years; mean PASI score 18.5 ± 7.5). Control samples were obtained from macroscopically unaffected skin surrounding moles from healthy individuals undergoing cosmetic surgery for mole removal (*n* = 7; female: male ratio 2:5; mean age 49 ± 26 years).

### Mice

All animal experiments were approved by the 2nd Local Institutional Animal Care and Use Committee (IACUC) in Kraków (approval nos. 320/2018 and 80/2021) and were performed in compliance with national and European legislation on animal welfare. *Dgat1* knockout mice (B6.129S4-Dgat1^tm1Far^/J; strain no. 003824) were obtained from The Jackson Laboratory and maintained under specific pathogen-free conditions at the animal facility of the Faculty of Biochemistry, Biophysics and Biotechnology, Jagiellonian University, Kraków. Sex-matched 8-week-old littermates were used in all experiments. Both male and female mice were used in all experiments to account for sex as a biological variable and to increase the rigor and translational relevance of the study findings. Data obtained from male and female mice were pooled for statistical analyses.

### Mouse keratinocyte isolation and culture

Primary keratinocytes were isolated from neonatal (0–1 day) WT or *Dgat1*KO murine back skin. Immediately after dissection, skin samples were placed in ice-cold dispase digestion buffer (4 mg/mL in PBS; Gibco, Thermo Fisher Scientific) and incubated overnight at 4 °C. The epidermal layer was then separated and incubated in TrypLE Select solution (Gibco, Thermo Fisher Scientific) for 10 min, followed by neutralization with CnT-07 medium (CELLnTEC) and gentle mechanical dissociation to obtain single cells. Keratinocytes were pelleted by centrifugation (5 min, 180 g), resuspended in CnT-07 medium (CELLnTEC) supplemented with 10 µM ROCK inhibitor, and cultured at 37 °C in a humidified 5% CO₂ incubator. Culture medium was replenished every 2–3 days. Upon reaching ~ 80% confluence, cells were seeded into 24- or 96-well plates at a density of 5 × 10⁴ cells/cm².

### Proliferation assays

Primary keratinocytes derived from WT and *Dgat1*KO mice were seeded in 96-well plates. After a 24-h attachment period, the culture medium was replaced and cells were treated with 200 nM all-*trans* retinoic acid (atRA). Proliferation was assessed using the Cell Proliferation ELISA BrdU assay (Roche) according to the manufacturer’s instructions, with measurements taken at 72 h. Cell death and relative cell viability were assessed using the CellTox™ Green Cytotoxicity Assay (Promega) according to the manufacturer’s instructions, with modifications. Fluorescence was measured as relative fluorescence units (RFU) using a plate reader. For each experimental condition, six replicate wells were prepared. At the endpoint of the experiment, three wells were treated with a lysis solution to induce complete cell lysis, while the remaining three wells were left untreated. Subsequently, CellTox™ Green reagent was added to all wells. Fluorescence measured in non-lysed wells was used as a readout of cell death and is presented as RFU. In parallel, lysed wells provided an internal reference corresponding to the total cellular DNA content (maximal fluorescence signal), allowing normalization for differences in cell number between conditions. Relative cell viability was calculated by subtracting the RFU of non-lysed wells from the RFU of fully lysed wells within each condition.

### RNA-seq – sample preparation

Back skin from 8-week-old WT and *Dgat1*KO mice was harvested and digested overnight in ice-cold dispase buffer (4 mg/mL in PBS). Owing to the high abundance of RNases in mouse epidermis, special precautions were taken to preserve RNA integrity. Following digestion, the epidermal layer was peeled off, transferred into Fenozol Plus lysis solution, and homogenized mechanically with zirconia/silica beads using the Bead-Beat Total RNA Mini Kit (A&A Biotechnology). After bead removal, chloroform was added at a 1:5 ratio, and samples were centrifuged at 12,000 g for 20 min. The aqueous phase was collected, mixed with isopropanol at a 1:2 ratio, and transferred to RNeasy Fibrous Tissue Mini Kit (Qiagen) spin columns. RNA isolation and purification, including DNase treatment, were performed according to the manufacturer’s protocol. Purified RNA was shipped to CeGaT GmbH (Tübingen, Germany) for further processing, including additional purification, library preparation using the KAPA RNA HyperPrep Kit with RiboErase (HMR) (Roche), and sequencing on the NovaSeq 6000 platform (2 × 100 bp).

### RNA-seq – analysis

Demultiplexing of sequencing reads was performed with Illumina bcl2fastq (2.20) and adapters were trimmed with Skewer (0.2.2) [17]. The quality of FASTQ files was analyzed with FastQC (0.12.1) (https://www.bioinformatics.babraham.ac.uk/projects/fastqc/*)* and MultiQC (1.28) [18] before alignment to mm39 mouse genome assembly using HISAT2 (2.2.1) [19]. Raw gene count matrices were created with featureCounts (Rsubread v2.20.0) using build-in mm39 annotation [20] and differential expression analysis was carried out with DESeq2 (1.46.0) [21]. Functional enrichment analyses were performed using DAVID web tool (v2024q4) [22] and GSEApy software (1.1.10) [23] with MSigDB (v2025.1.Mm) [24–26] and external reference rexinoid gene signatures [27]. Enrichment results were visualized as interaction networks using Cytoscape (v3.9.1) [28]. Gene regulatory network analysis was performed using VIPER (1.40.0) [29] with DoRothEA (1.18.0) TF-target collection [30].

### Single cell sequencing data analysis

Single-cell RNA sequencing (scRNA-seq) data from imiquimod (IMQ)-treated mouse dorsal skin were obtained from publicly available datasets: GSE165021 (GSM5024746, GSM5024747, GSM5024748, GSM5024749) [31], GSE193350 (GSM5795800) and GSE197803 (GSM5795802) [32], as well as GSE238086 (GSM7658353, GSM7658354) [33]. Raw data (Cell Ranger outputs: matrices, barcodes, and features) were processed using the Scanpy framework [34]. Ambient RNA contamination was corrected using DecontX [35], and doublets were identified and removed using scDblFinder [36].

Quality control was performed using a median absolute deviation (MAD)-based approach (5 MAD), applied to total counts, number of detected genes per cell, and mitochondrial transcript content. The data were subsequently normalized and subjected to dimensionality reduction, nearest-neighbor graph construction, and visualization within Scanpy. Batch correction and dataset integration were performed using BBKNN [37]. Clustering was conducted using the Leiden algorithm [38], and cell type annotation was guided by a combination of CellTypist automated predictions [39] and manual curation based on established marker genes.

### Psoriasis-like skin dermatitis induction and tissue preparation

Psoriasis-like dermatitis was induced using the imiquimod (IMQ) model as previously described [40]. Eight-week-old mice were selected for all experiments, as skin alterations are particularly dynamic beyond this age [6]. Mice received topical applications of 15 mg Aldara™ cream (5% IMQ; Meda AB) or Vaseline (Unilever) twice daily for up to 6 days on 1 cm² shaved and depilated dorsal skin.

Disease severity was assessed daily using a modified Psoriasis Area and Severity Index (PASI), in which erythema, scaling, and lesion area were scored independently on a scale from 0 (none) to 4 (very marked). Transepidermal water loss (TEWL) was measured on IMQ-treated skin using a Tewameter TM300 (Courage + Khazaka Electronic). At the end of treatment, mice were euthanized by anesthetic overdose, and skin tissues were collected.

Skin biopsies (0.5 cm²) were divided into two fragments: one was embedded in cryo-matrix, snap-frozen in liquid nitrogen vapor, and stored at − 80 °C for histological and immunohistochemical analyses; the other was immersed in Fenozol Plus and frozen at − 80 °C for subsequent qPCR analyses.

### Histochemistry

Murine 10-µm frozen skin sections were fixed in ice-cold acetone and stained with hematoxylin and eosin (H&E; Thermo Fisher Scientific). Images were acquired using a Nikon Eclipse light microscope. Epidermal thickness was quantified manually with NIS-Elements software (Nikon). For measurements, the epidermis was defined as the region extending from the basement membrane, including stratum basale keratinocytes, to the last visible nucleated layer. For each mouse, up to 100 measurements were performed, and the mean value was plotted as a single data point.

### Immunofluorescence

Frozen 10-µm skin sections from WT and *Dgat1*KO mice were fixed in ice-cold acetone and incubated with rat anti-mouse Ki67 primary antibodies (BioLegend), followed by goat anti-rat APC-conjugated secondary antibodies (Jackson ImmunoResearch). Isotype controls were performed using rat IgG2a κ. Nuclei were counterstained with Hoechst 33,258 (Invitrogen), and sections were mounted with Fluorescence Mounting Medium (Dako). Images were acquired using a fully motorized Nikon Eclipse fluorescence microscope and analyzed with NIS-Elements software (Nikon).

### Fluorescence image analysis

Fluorescence images were analyzed using Fiji (ImageJ, version 1.54p) as previously described [41], with minor modifications. All images were acquired using fixed imaging parameters. Regions corresponding to the epidermis or hair follicles were manually delineated as regions of interest (ROIs) using the freehand selection tool. Mean fluorescence intensity within each ROI was measured directly on raw images.

### real-time qPCR

Total RNA was extracted from primary keratinocyte cultures, murine skin biopsies, and human skin biopsies using Fenozol Plus and the Total RNA Zol-Out Kit (A&A Biotechnology). RNA concentration and purity were assessed with a NanoDrop 1000 spectrophotometer (Thermo Fisher Scientific). cDNA was synthesized using NxGen M-MulV reverse transcriptase (Lucigen) with random primers (Promega) on a Labcycler thermocycler (SensoQuest).

Real-time quantitative PCR was performed on a CFX96 thermocycler (Bio-Rad Laboratories) using RT HS PCR Mix SYBR (A&A Biotechnology) and gene-specific primers (Genomed) listed in Table 1. Each sample was analyzed in duplicate. Mouse mRNA expression was normalized to the geometric mean of housekeeping genes (*Gapdh*, *Ppia*, *B2m*), and human *DGAT1* expression was normalized to *GAPDH*. Relative expression levels were calculated using the 2^–ΔΔCt^ method [42].

**Table 1:** Primer sequences for mouse and human genes used in quantitative real-time PCR (qPCR)

Symbol	Name	Forward primer sequence	Forward primer sequence
*GAPDH*	GAPDH	GAGTCAACGGATTTGGTCGTATTG	GAGTCAACGGATTTGGTCGTATTG
*DGAT1*	DGAT1	ATCCTTGAGATGCTGTTCTTCACC	ATGATGCGTGAGTAGTCCATGTC
*Gapdh*	GAPDH	TGTGTCCGTCGTGGATCTGA	TTGCTGTTGAAGTCGCAGGAG
*B2m*	beta2-microglobulin	GGACTGGTCTTTCTATATCCTGGC	GATCACATGTCTCGATCCCAGTAG
*Ppia*	cyclophilin A	AGCATACAGGTCCTGGCATCTTGT	CAAAGACCACATGCTTGCCATCCA
*Dgat1*	DGAT1	TGTGTGGTGATGCTGATCCTGAGT	GCCAGGCGCTTCTCAATCTGAAAT
*Krt1*	keratin 1	AGAACAAGCTGAATGAGATAGAGGA	GCACTCTCCAGACATCCTGA
*Krt14*	keratin 14	CAGCAGAACCAGGAGTACAAAATC	AGAATTGGGAAGATGAAAGGTGGG
*Ivl*	involucrin	ATTGGAAGAGAAGCAGCATCAGAA	CTCTTCCAGATCCTCTGCCATATC
*Lor*	loricrin	CAAGGGTGTGCCAGTCTGC	TGCAACCCGGTGACCTTAC
*Crbp1*	cellular retinol-binding protein 1	GATGAACTTCACCTGGAAATGAGA	GGGCTGCTCAGTGTACTTTCTTAA
*Crabp2*	cellular retinoic acid-binding protein 2	TGGGAGACCCTGTAAGAGTT	TGGTGCACCAACGTCATCT

### Statistical analysis

Statistical analyses were performed using GraphPad Prism 9 (GraphPad Software). Data are presented as mean ± standard error of the mean (SEM). Details of the statistical tests applied and the number of samples per group are provided in the figure legends.

## Results

### DGAT1 deficiency drives keratinocyte hyperproliferation and barrier dysfunction

Previously published data show that DGAT1 deficiency affects skin function, causing abnormalities in fur lipids, sebaceous gland atrophy, hair loss and disrupting the epidermal barrier [4–6]. To extend these findings at the transcriptional level, we focused on epidermis and profiled keratinocytes from WT and *Dgat1*KO mice using RNA sequencing (bulk RNA-seq). During our current work, we observed enhanced pigmentation and disturbed hair cycles in most *Dgat1*KO mice immediately after reaching puberty, which corresponded with previously published data [6]. In order to avoid confounding age-related factors, for RNA-seq and subsequent analyses, we selected 8-week-old mice without visible skin abnormalities.

**Fig. 1:**
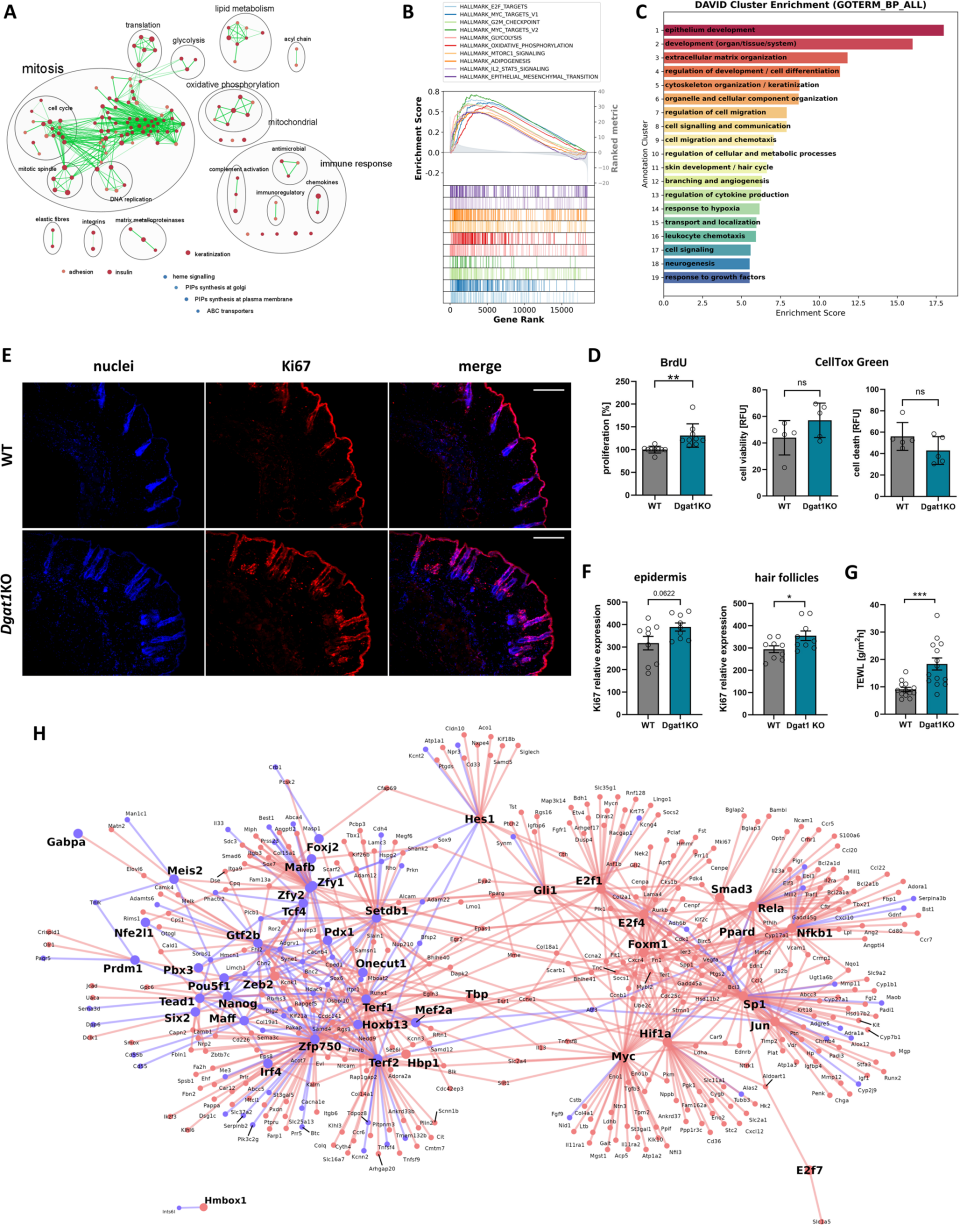
Lack of DGAT1 results in keratinocytes hyperproliferation. Bulk RNA-seq was performed on keratinocytes isolated from WT and *Dgat1*KO mice. **(A)** Pathway enrichment analysis. Gene set enrichment analysis (GSEA) visualized with Cytoscape Enrichment Map App. Each node (circle) corresponds to a gene set either up-regulated (red) or down-regulated (blue) in response to DGAT1 deficiency. The fill color gradient represents the p-value-corrected enrichment score. Edges (green lines) link sets with shared genes, and clusters of gene sets are outlined with black circles and annotated. FDR < 0.05. **(B)** GSEA plots with top 10 MH (mouse-ortholog hallmark) gene sets enriched in *Dgat1*KO epidermis compared to WT. **(C)** DAVID Gene Ontology: Biological Process terms enriched among gene clusters in *Dgat1*KO epidermis, top 20 processes with Enrichment Score > 5.00 and p-adj < 0.01. **(D)** In vitro proliferation (BrdU) and cytotoxicity (CellTox Green) assays measured in primary mouse keratinocytes cultured for 72 h. The data are shown as a mean ± SEM; ** *p* < 0.01, *** *p* < 0.001 by *t*-test. Gray bars = WT mice; turquoise bars = *Dgat1*KO mice. **(E)** Representative images of Ki67 protein expression in WT and *Dgat1*KO mouse skin. Skin sections were stained for Ki67 (red) and DNA (blue). Ki67^+^ cells indicate proliferating cells. Data come from one experiment and are representative of at least four experiments. Scale bar = 200 μm. **(F)** Mean fluorescence intensity for Ki67 staining was measured in skin sections from WT and *Dgat1*KO mice. The data are shown as a mean ± SEM; * *p* < 0.05 by *t*-test. Gray bars = WT mice; turquoise bars = *Dgat1*KO mice. **(G)** Transepidermal water loss (TEWL) was measured in WT and *Dgat1*KO mice. The data are shown as a mean ± SEM; *** *p* < 0.001 by *t*-test. Gray bars = WT mice; turquoise bars = *Dgat1*KO mice. **(H)** Gene regulatory network (GRN) analysis. Each node (circle) corresponds to a transcription factor either up-regulated (red) or down-regulated (blue) in response to DGAT1 deficiency

The pairwise gene comparison indicated that the expression of 1222 and 520 transcripts was significantly up- or downregulated (p-adjusted < 0.01, |fold change| > 2.0), respectively, in *Dgat1*KO epidermis compared to WT (Supplementary Figure 1). Functional analysis by Gene Ontology (GO) enrichment annotation revealed that the genes with upregulated expression in *Dgat1*KO mice were mainly assigned to groups related to keratinocyte proliferation and inflammatory responses (Fig. 1A-C). Other pathways found to be elevated in the *Dgat1*KO epidermis were related to mitochondrial respiration/oxidative phosphorylation, lipid metabolism, glycolysis, as well as general terms associated with epithelium development, cell differentiation, tissue remodeling, hypoxia and hair cycle.

Hyperproliferation of *Dgat1*KO keratinocytes was confirmed by in vitro proliferation assay using primary keratinocytes isolated from WT and *Dgat1*KO mouse, without accompanying changes in cell death (Fig. 1D). Interestingly, Ki67 staining in mouse skin sections shows increased keratinocytes’ proliferation not only in the epidermis, but also even more pronounced in hair follicles (Fig. 1E, F). Furthermore, keratinocyte hyperproliferation is accompanied by skin barrier dysfunctions, observed as increased transepidermal water loss (TEWL) measurements, suggesting impaired epidermal permeability in *Dgat1*KO mice (Fig. 1G).

To further investigate the impact of DGAT1 deficiency on gene expression changes in keratinocytes, we performed a gene regulatory network (GRN) analysis, which enables the identification of interactions between transcription factors and the genes they regulate [43]. In DGAT1-deficient cells, we observed marked upregulation of the downstream targets of transcription factors Hif1a, Jun, Myc, E2f1, E2f4, Foxm1, Gli1, Hes1 (Fig. 1H), all of which are known to be involved in the positive regulation of cell proliferation.

Together, these findings demonstrate that DGAT1 deficiency leads to pronounced keratinocyte hyperproliferation and barrier dysfunction, establishing a basis to further explore its role in pathological conditions characterized by excessive epidermal growth.

### DGAT1 expression is elevated in psoriatic skin keratinocytes and modulates disease progression

**Fig. 2:**
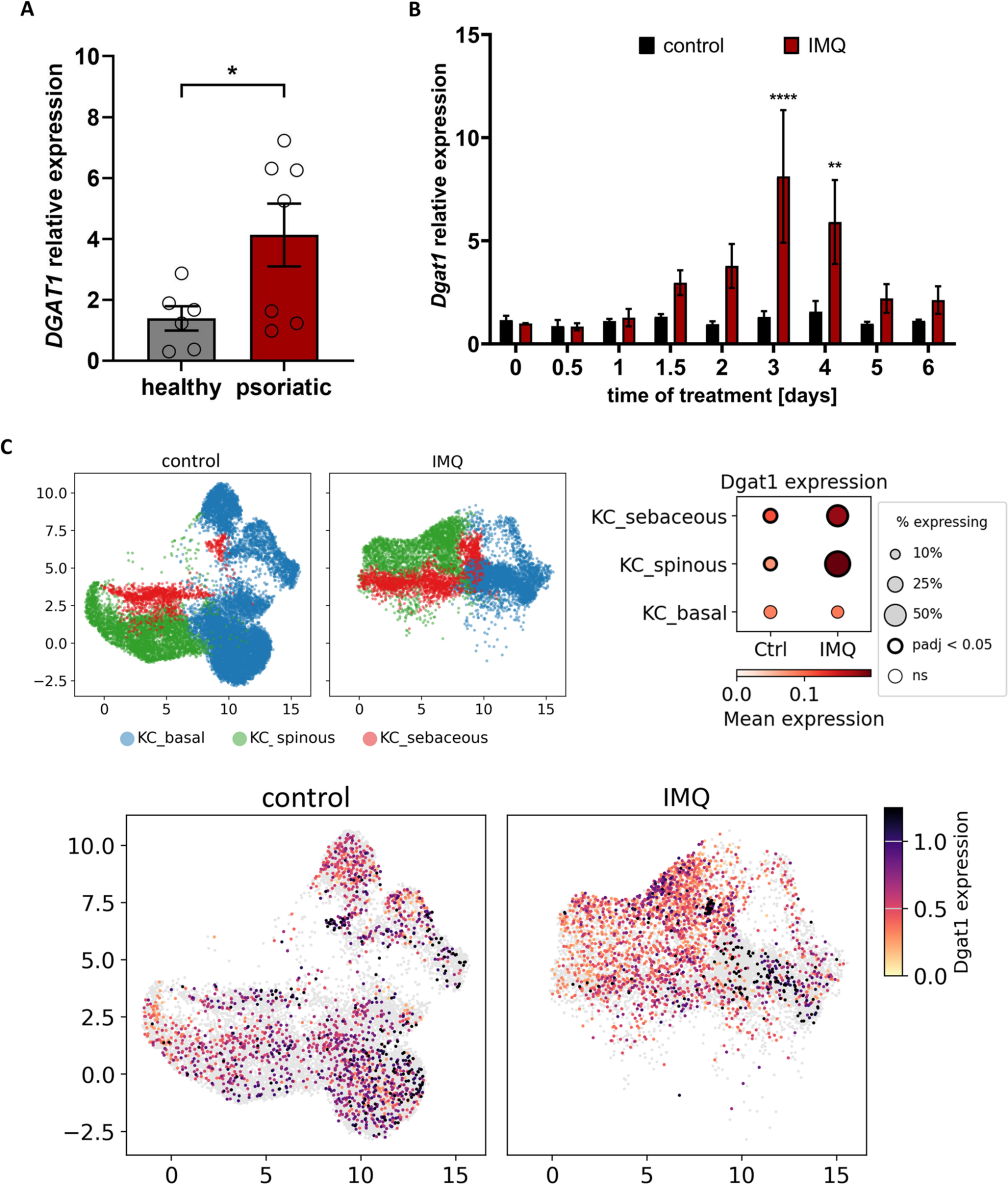
**DGAT1 transcript levels are increased in the lesional skin of patients with psoriasis and in the experimental model of psoriasis. (A)** *DGAT1* mRNA expression was measured in the skin biopsies from the indicated human donors by real-time qPCR. The data are shown as a mean ± SEM; * *p* < 0.05 by *t*-test. **(B)** C57BL/6J mice were subjected to an IMQ-based experimental model of psoriasis. The skin was harvested at the days indicated and subjected to real-time qPCR analysis for *Dgat1* mRNA expression. The data are shown as a mean ± SEM; ** *p* < 0.01, **** *p* < 0.0001 by two-way ANOVA, Tuckey *post hoc* test. (**C**) *Dgat1* gene expression across keratinocyte subsets in imiquimod (IMQ)-treated mouse dorsal skin. UMAP visualization of the integrated scRNA-seq datasets colored according to keratinocyte subclustering with finely annotated subtypes (upper left panel). Dot plot summarizing *Dgat1* expression across keratinocyte subtypes, where dot size represents the fraction of expressing cells, and color intensity indicates normalized *Dgat1* expression levels; bold outlines denote statistically significant differences between corresponding conditions, i.e., control and IMQ-treated samples (Wilcoxon rank-sum test, adjusted p-value < 0.05) (upper right panel). Feature plots showing normalized *Dgat1* expression across keratinocyte subclusters (lower panel)

Our observation that DGAT1 profoundly regulates keratinocyte proliferation prompted us to examine its role in a pathological context of hyperproliferation. Alterations in barrier function are associated with a number of skin diseases, including psoriasis [44]. As a hallmark of psoriasis is excessive keratinocyte proliferation, we investigated whether *DGAT1* gene expression is altered during the disease. Therefore, we collected skin biopsies from psoriasis patients and healthy controls and analyzed *DGAT1* gene expression. Interestingly, *DGAT1* transcript levels were fourfold higher in lesional skin than in healthy donor skin (Fig. 2A) and were also elevated in the skin of mice in an experimental psoriasis model (Fig. 2B).

To further investigate the cell type-specific regulation of *Dgat1* in the context of psoriatic-like inflammation, we analyzed publicly available single-cell RNA sequencing (scRNA-seq) datasets derived from the skin of control and imiquimod (IMQ)-treated mice [31–33]. This analysis revealed that keratinocytes – specifically those annotated as sebaceous and spinous subpopulations – represent the only skin cell population exhibiting a statistically significant upregulation of *Dgat1* expression in response to IMQ treatment (Fig. 2C). These findings indicate that *Dgat1* induction is restricted to defined keratinocyte subsets under inflammatory conditions, suggesting a cell type-specific role for DGAT1 in epidermal remodeling associated with psoriasis-like pathology.

**Fig. 3:**
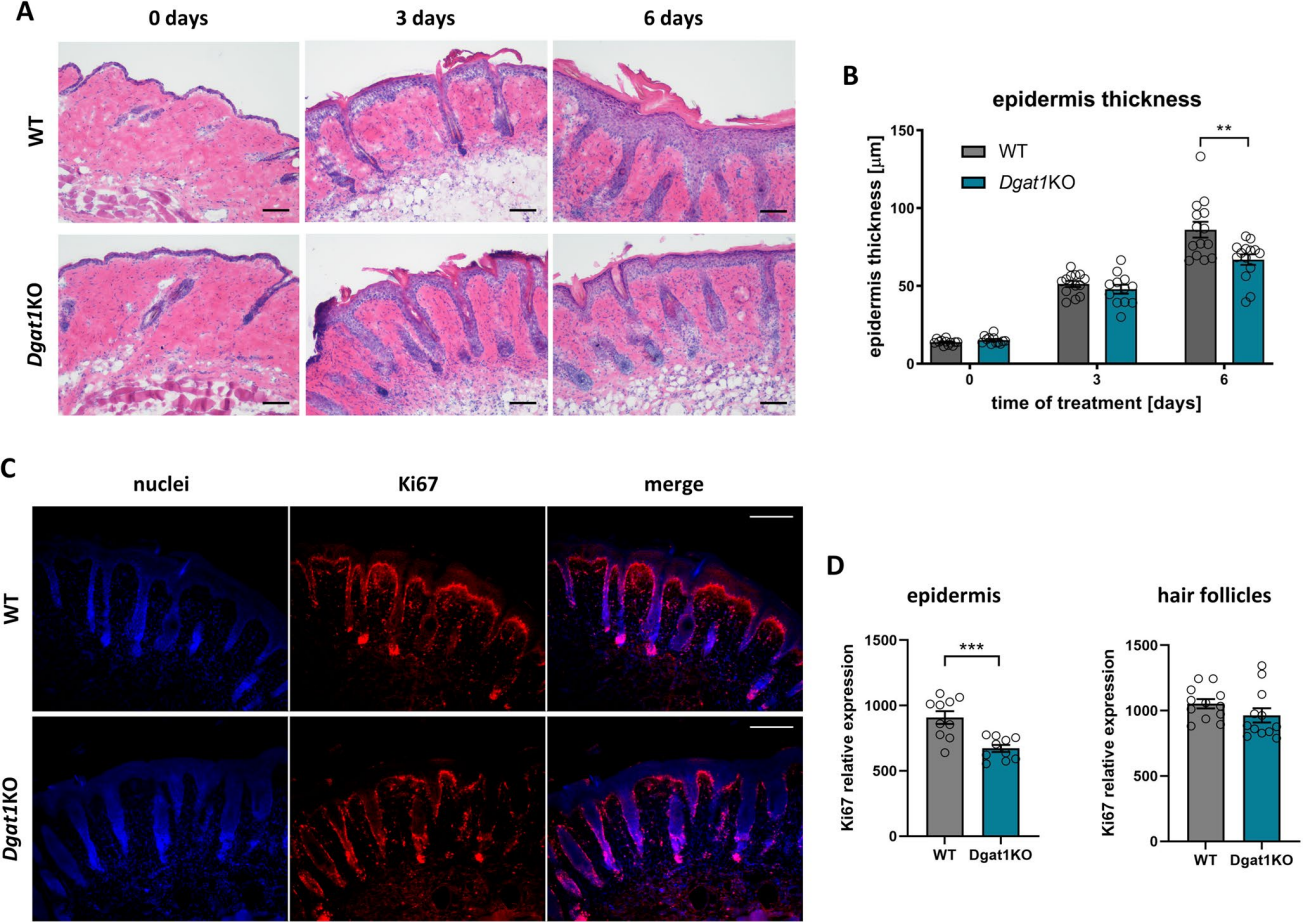
DGAT1 reduces the thickening of the epidermis and keratinocyte proliferation in a mouse model of psoriasis. WT and *Dgat1*KO mice were treated with IMQ to induce psoriasis-like dermatitis. **(A)** Representative images of murine skin histology. The skin was harvested at the days indicated and analyzed using histology. Results are representative of at least four independent experiments. Scale bar = 100 μm. **(B)** Epidermis thickness was measured in skin sections from WT and *Dgat1*KO mice. The data are shown as a mean ± SEM; ** *p* < 0.01 by two-way ANOVA, Tuckey *post hoc* test. Gray bars = WT mice; turquoise bars = *Dgat1*KO mice. **(C)** Representative images of Ki67 protein expression in WT and *Dgat1*KO mouse skin after 6 days of IMQ treatment. Skin sections were stained for Ki67 (red) and DNA (blue). Ki67^+^ cells indicate proliferating cells. Data come from one experiment and are representative of at least four experiments. Scale bar = 200 μm. **(D)** Mean fluorescence intensity for Ki67 staining was measured in skin sections from WT and *Dgat1*KO mice. The data are shown as a mean ± SEM; *** *p* < 0.001 by *t*-test. Gray bars = WT mice; turquoise bars = *Dgat1*KO mice

Based on these findings, we hypothesized that DGAT1 deficiency would further enhance keratinocyte proliferation under psoriatic conditions, similar to what we observed in untreated DGAT1-deficient mice. Surprisingly, despite the increased basal proliferation in *Dgat1*KO mice, treatment with IMQ resulted in reduced epidermal thickness by day 6 (Fig. 3A, B). This reduction was accompanied by diminished keratinocyte proliferation, as shown by decreased relative expression of Ki67 (Fig. 3C, D), specifically within the basal layer of the epidermis. In contrast, Ki67 expression levels in hair follicles remained comparable between WT and *Dgat1*KO mice. A further indication of reduced proliferation was the downregulation of *Krt14*, a marker of proliferative keratinocytes, in IMQ-treated *Dgat1*KO skin (Supplementary Figure 2). Importantly, during IMQ treatment (days 3 and 6), no significant changes were observed in the expression of other genes associated with keratinocyte proliferation or differentiation (Supplementary Figure 2). These findings suggest that the relationship between keratinocyte proliferation, differentiation, and epidermal remodeling is dynamic and may vary depending on the stage of IMQ-induced skin inflammation.

**Fig. 4:**
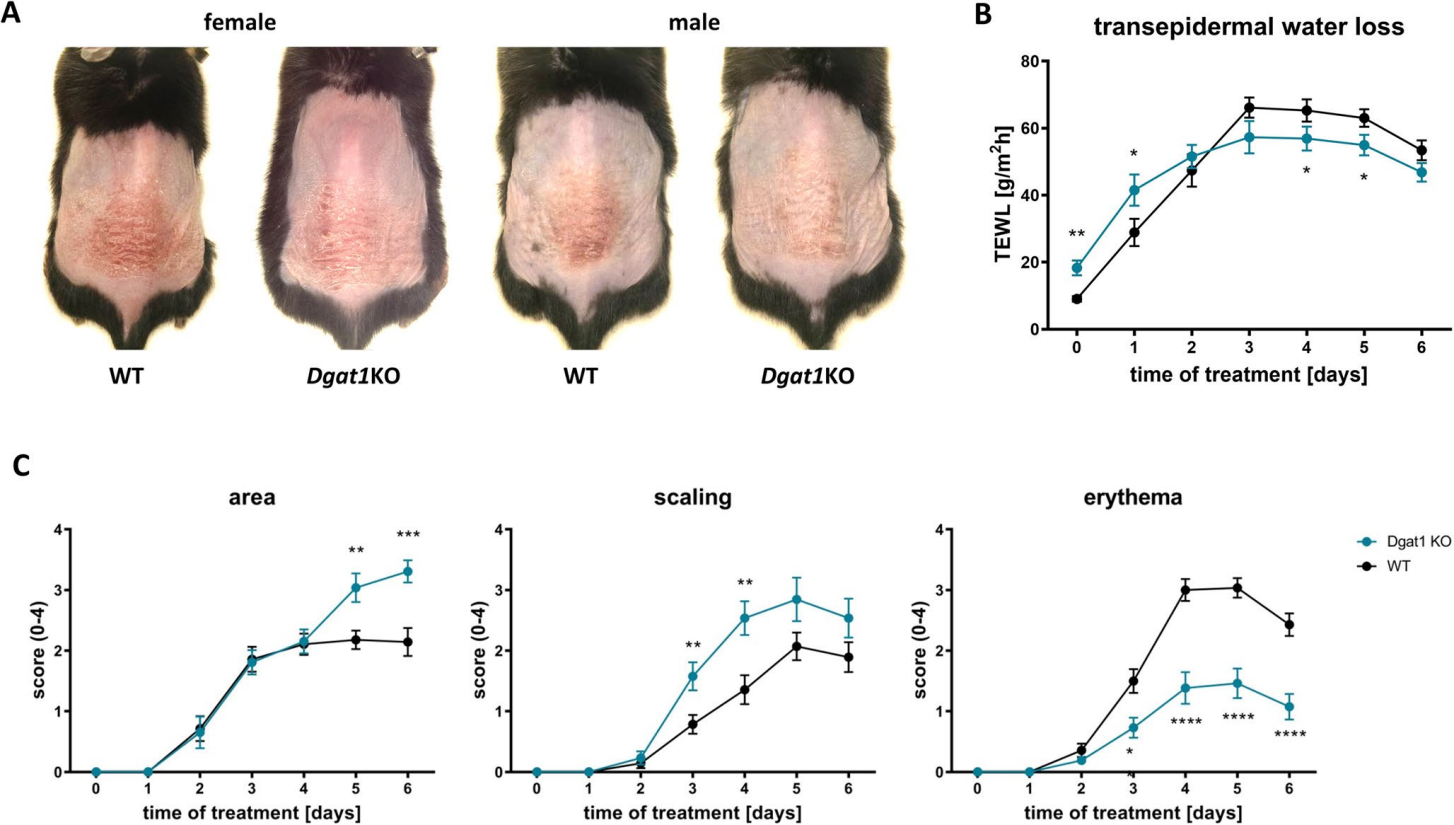
DGAT1 affects the course of psoriasis in an IMQ-driven experimental model. WT and *Dgat1*KO mice were treated with IMQ to induce psoriasis-like dermatitis. **(A)** The representative images of WT and *Dgat1*KO lesional skin after 6 days of IMQ treatment. **(B)** Transepidermal water loss (TEWL) was measured in WT and *Dgat1*KO mice (*n* = 14). The data are shown as a mean ± SEM; * *p* < 0.05, ** *p* < 0.01, by two-way ANOVA, Tuckey *post hoc* test. **(C)** Cumulative data showing the size of the lesion, scaling, and erythema (*n* = 14). The data are shown as a mean ± SEM; * *p* < 0.05, ** *p* < 0.01, *** *p* < 0.001, **** *p* < 0.0001 by two-way ANOVA, Tuckey *post hoc* test

Interestingly, DGAT1 deficiency also affected disease progression at the tissue level. In our previously published data, we demonstrated that DGAT1 deficiency leads to reduced neutrophil recruitment to the lesional skin in a psoriasis-like model [45]. Importantly, the lesser infiltration to the skin of *Dgat1*KO mice by neutrophils was a combined effect of the reduced migratory capacity of neutrophils lacking DGAT1 as well as the less-supportive cutaneous environment of *Dgat1*KO mice. Here, we observed that while TEWL and skin erythema gradually decreased in *Dgat1*KO mice, scaling and the expansion of psoriatic lesion areas were markedly increased compared to WT controls (Fig. 4A–C). These findings indicate that DGAT1 has context-dependent effects on keratinocyte biology, promoting proliferation under homeostatic conditions but restraining it in an inflammatory, hyperproliferative environment such as psoriasis. Considering the markedly increased scaling observed in *Dgat1*KO mice, we speculate that the reduced epidermal thickness may result not only from decreased keratinocyte proliferation, but also from accelerated keratinocyte turnover and enhanced desquamation. However, this possibility requires further investigation in future studies.

Thus, DGAT1 emerges as a context-dependent regulator of keratinocyte proliferation, enhancing growth under homeostatic conditions while limiting it in psoriatic inflammation, prompting us to investigate the underlying molecular mechanisms.

### DGAT1 deficiency enhances RA signaling in keratinocytes

Given the non-conclusive results, we next sought to elucidate the molecular mechanisms governing keratinocyte regulation under homeostatic conditions. Since most of the skin abnormalities in DGAT1-deficient mice have been attributed to elevated levels of retinoic acid (RA) – with DGAT1 deficiency shown to lead to elevated RA levels in the skin [6, 7], and DGAT1-deficient skin exhibiting increased sensitivity to topically applied retinol. We therefore investigated this pathway in our study.

**Fig. 5:**
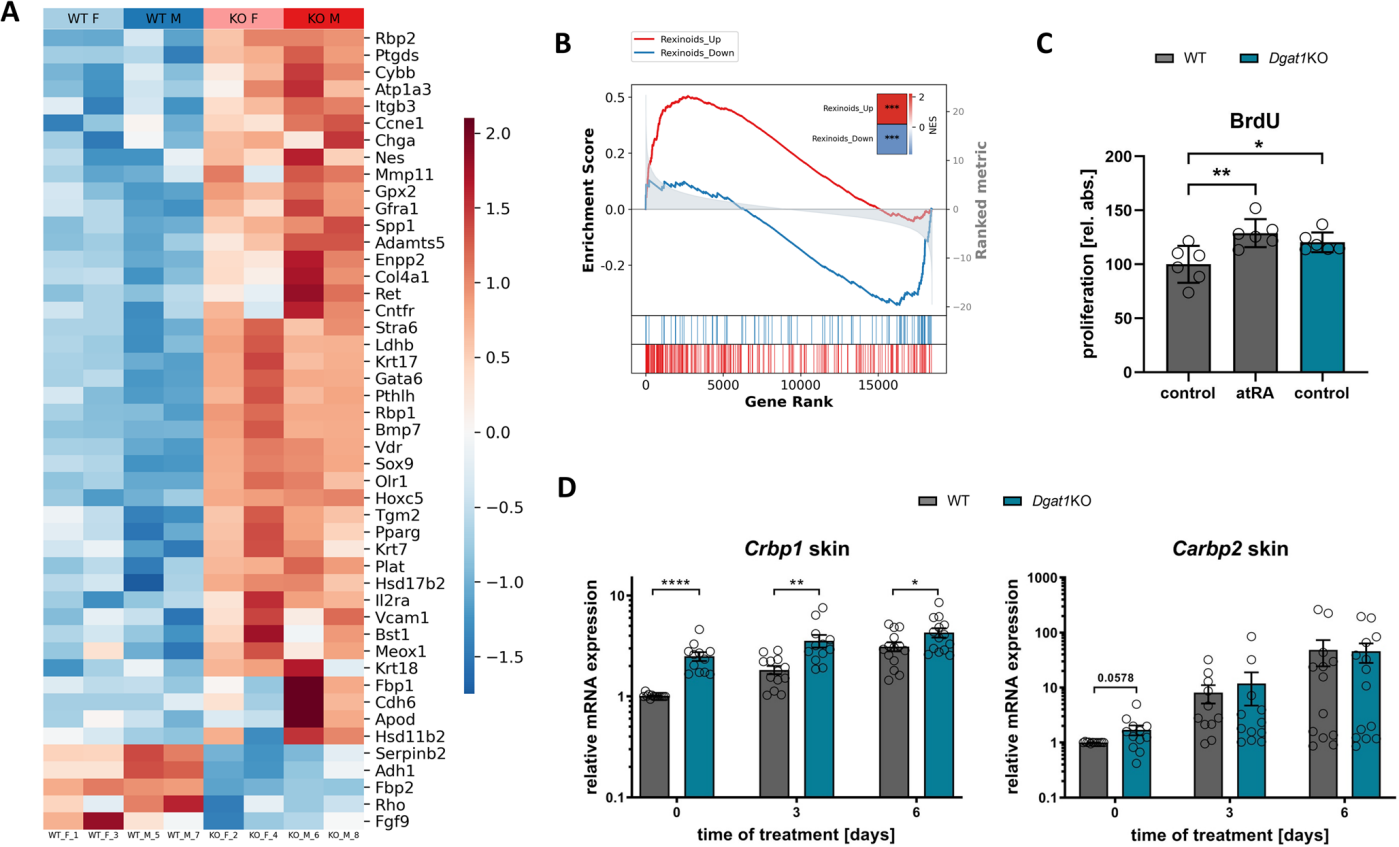
Lack of DGAT1 results in upregulation of RA-targeted genes. Bulk RNA-seq was performed on epidermis isolated from WT and *Dgat1*KO mice (*n* = 4). **(A)** Heatmap of differentially expressed RA-targeted genes [62]. **(B)** GSEA plots and heatmap showing the significant changes in retinoids-responsive gene signature in *Dgat1*KO epidermis (both up- and down-regulated RXR-RAR gene signature) [27]. **(C)** In vitro proliferation (BrdU) assays measured in primary mouse keratinocytes cultured by 72 h with or without all-*trans* retinoic acid (atRA). The data are shown as a mean ± SEM; * *p* < 0.05, ** *p* < 0.01 by *t*-test. Gray bars = WT mice; turquoise bars = *Dgat1*KO mice. **(D)** Relative expression of genes coding RA binding proteins, during IMQ-treatment of WT and *Dgat1*KO mice. The data are shown as a mean ± SEM; * *p* < 0.05, ** *p* < 0.01, **** *p* < 0.0001 by two-way ANOVA, Tuckey *post hoc* test. Gray bars = WT mice; turquoise bars = *Dgat1*KO mice

Our RNA-seq analysis revealed that absence of DGAT1 causes a strong increase in known RA-target genes expression in the murine epidermis (Fig. 5A). Additionally, we acquired gene signatures from retinoid-treated keratinocytes from publicly available study, which we then used as a reference for enrichment analysis [27]. This approach showed that both up- and down-regulated genes that characterize retinoid signaling are significantly enriched in DGAT1 deficient epidermis (Fig. 5B).

The role of elevated RA levels in the absence of DGAT1 in modulating keratinocyte proliferation was further supported by in vitro experiments using murine keratinocytes treated with all-*trans* retinoic acid (atRA). Proliferation of atRA-stimulated WT keratinocytes increased to levels comparable to those observed in untreated *Dgat1*KO keratinocytes (Fig. 5C). Moreover, RA is known to tightly regulate its own bioavailability in the epidermis by modulating the expression of enzymes involved in RA metabolism as well as proteins responsible for its intracellular delivery and sequestration [46, 47]. Consistent with this, our qPCR analysis demonstrated increased expression of *Crbp1* (2.5-fold) and *Crabp2* (1.7-fold) – genes encoding RA-binding proteins whose transcription is induced by elevated free RA levels [6] – in the skin of *Dgat1*KO mice (Fig. 5D). Notably, the increased expression of these genes in *Dgat1*KO mice was attenuated over the course of IMQ treatment, suggesting a shift in RA availability under inflammatory conditions.

Together, these data suggest that dysregulated RA signaling represents one of the central mechanisms linking DGAT1 deficiency to altered keratinocyte proliferation, providing a plausible molecular basis for its effects in skin homeostasis and disease.

## Discussion

DGAT1 has emerged as a critical regulator of retinoid homeostasis by functioning as an acyl-CoA: retinol acyltransferase (ARAT), converting retinol into retinyl esters and preventing excessive RA accumulation [6, 7]. In the absence of DGAT1, retinol that enters keratinocytes cannot be efficiently esterified and stored as retinyl esters. Instead, excess retinol is converted to retinaldehyde and subsequently to retinoic acid (RA), leading to its accumulation and modulation of gene expression [48], which could have major consequences for keratinocyte behavior and skin barrier function. In our previous work, we demonstrated that DGAT1 deficiency profoundly affects psoriasis pathogenesis by reducing neutrophil infiltration into lesional skin and impairing neutrophil migration [45]. These findings highlighted DGAT1 as a regulator of immune cell recruitment and inflammatory responses in the skin.

Building on this, the present study was designed to address whether DGAT1 also regulates keratinocyte biology, thereby influencing epidermal homeostasis and hyperproliferative diseases such as psoriasis. Specifically, we sought to define the transcriptional, cellular, and molecular consequences of DGAT1 deficiency in keratinocytes under both physiological and inflammatory conditions, with particular emphasis on its interplay with RA signaling pathways.

In this study, we demonstrate for the first time a profound impact of DGAT1 on keratinocyte proliferation. Surprisingly, this effect was evident only under homeostatic conditions and not in psoriasis. We postulate RA as a key molecular mediator regulating keratinocyte proliferation. Altered expression of retinoid-related proteins and receptors has been previously reported in psoriatic skin [14, 49, 50], and transcriptomic analyses revealed that great proportion of psoriasis-associated genes are also regulated by RA and more than 60% of them are regulated in opposite directions by RA and in psoriasis [15]. These findings strongly support the notion of dysregulated RA signaling in the disease. Consistent with this, we observed increased DGAT1 gene expression in both the murine psoriasis model and patient lesions. Importantly, analysis of publicly available single-cell RNA sequencing data revealed that this upregulation of *Dgat1* in response to psoriatic-like inflammation is driven predominantly by keratinocytes.

The opposing effects of DGAT1 deficiency on keratinocyte proliferation in homeostasis and psoriasis likely reflect the context-dependent activity of RA, a phenomenon widely reported in dermatological research. While RA can stimulate keratinocyte proliferation in normal skin [15, 51], in hyperproliferative states such as psoriasis it promotes differentiation and suppresses proliferation [8, 52, 53]. Our results fit this model: DGAT1 loss increased keratinocyte proliferation in normal skin, but dampened proliferation in IMQ-induced lesions. Similar effects were reported in epidermal substitutes in vitro, where RA reduced epidermal thickness in psoriatic but not in healthy skin equivalents [54].

Beyond proliferation, RA also impacts epidermal barrier integrity. Increased transepidermal water loss (TEWL) in *Dgat1*KO mice indicates barrier dysfunction, consistent with classic observations that RA disrupts keratinocyte adhesion and alters stratification patterns [55, 56]. Excess RA levels in keratinocytes are a well-established driver of epidermal abnormalities and scaling – topically applied RA causes prominent scaling and increased corneocyte detachment through degradation of corneodesmosomes, with RA directly downregulating the expression of the corneodesmosomal cadherins DSG1 and DSC1, thereby altering the desquamation process [57]. Elevated RA further perturbs the protease–antiprotease balance [58], contributing to inflammation, erythema, and scaling [59] – features also observed in our *Dgat1*KO mice. These parallels raise the possibility that the cutaneous phenotype of DGAT1-deficient mice is, at least in part, attributable to excessive RA activity in the skin. Interestingly, despite the increased scaling observed in *Dgat1*KO mice during IMQ-induced psoriasis, other disease-associated parameters, including epidermal thickness, TEWL, erythema, and previously reported inflammatory cell infiltration [45], were reduced. This phenotype may reflect the complex effects of elevated RA signaling in the skin. Notably, clinically used retinoids such as acitretin and tazarotene are well known to initially induce irritation, erythema, dryness, and scaling, despite their therapeutic efficacy in psoriasis [60]. Thus, the increased scaling observed in *Dgat1*KO mice may coexist with attenuation of other pathological features of psoriasis and does not necessarily indicate exacerbated epidermal hyperproliferation or inflammation.

In this study, we provide novel evidence that DGAT1 critically regulates keratinocyte proliferation and barrier homeostasis, and we propose that this effect is mediated by elevated RA levels resulting from the absence of DGAT1. However, we acknowledge that direct functional evidence would be required to establish causality and to determine the cell-autonomous nature of the observed effects. Such approaches remain an important direction for future investigation. Furthermore, DGAT1 is a multifunctional enzyme with activity across several lipid metabolic pathways and multiple substrates [61], and it is likely that additional lipid-mediated mechanisms contribute to the complexity of its role in the skin. Understanding these interconnected pathways will require further study, particularly given the well-established reciprocal influence of RA on lipid metabolism itself. Collectively, our findings highlight a previously unrecognized link between DGAT1 activity and keratinocyte biology, offering new insight into the molecular mechanisms shaping epidermal function under both physiological and inflammatory conditions.

## Supplementary Information

Below is the link to the electronic supplementary material.

Supplementary figure 6 DGAT1-deficiency highly change the basal gene expression in the murine epidermis. Bulk RNA-seq performed on epidermis isolated from WT and Dgat1KO mice (n = 4). A) Principal component analysis B) Volcano plot with 20 most significantly changed genes labeled. C) Heatmap of differentially expressed genes (PNG 590 KB)

High Resolution Image (TIF 15.0 MB)

Supplementary figure 7 DGAT1 affects keratinocyte differentiation. Relative expression of genes characteristic for keratinocyte proliferation and differentiation in murine skin (Krt1 – keratin 1, Krt14 – keratin 14, Ivl – involucrin, Lor – loricrin). The data are shown as a mean ± SEM; * p < 0.05, ** p < 0.01 by t-test. Gray bars = WT mice; turquoise bars = Dgat1KO mice (PNG 582 KB)

High Resolution Image (TIF 15.0 MB)

## Data Availability

The raw and processed RNA-sequencing data generated during this study have been deposited in the European Nucleotide Archive (ENA) at EMBL-EBI under accession number PRJEB100543 (https://www.ebi.ac.uk/ena/browser/view/PRJEB100543). The data for this study have been deposited in the Scripts used for data analysis are available at https://github.com/kamila-kwiecien/DGAT1.
