# Supplementary figures and images for: DGAT1 regulates keratinocyte proliferation through the modulation of retinoid homeostasis
Source: J Physiol Biochem. 2026 Jun 10;82(1):58. doi: 10.1007/s13105-026-01196-w (PMC13253717; doi:10.1007/s13105-026-01196-w)

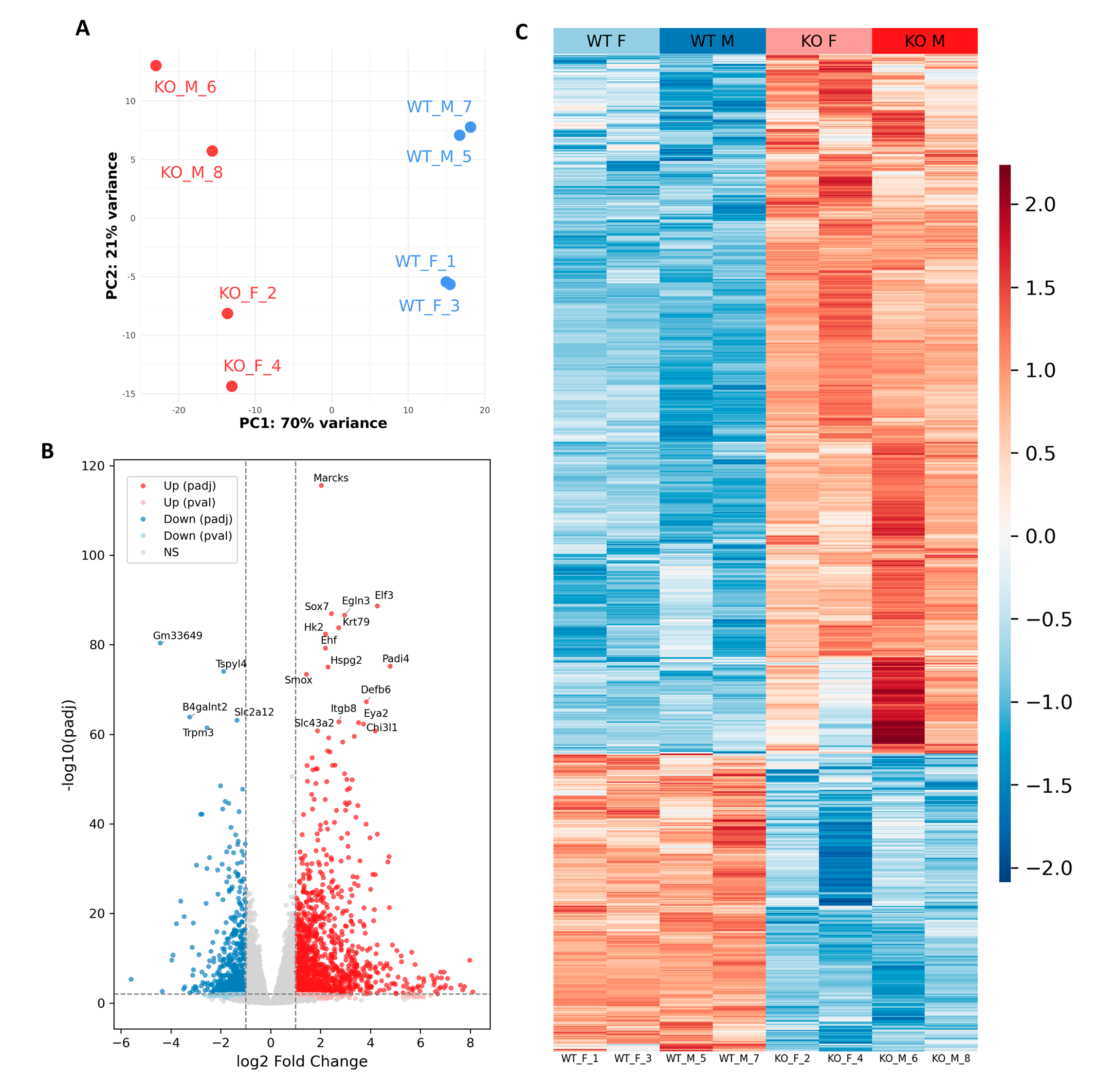

Supplement: Supplementary file 1 — DGAT1-deficiency highly change the basal gene expression in the murine epidermis. Bulk RNA-seq performed on epidermis isolated from WT and Dgat1KO mice (n = 4). A) Principal component analysis B) Volcano plot with 20 most significantly changed genes labeled. C) Heatmap of differentially expressed genes (PNG 590 KB) [file 13105_2026_1196_Fig6_ESM.png]

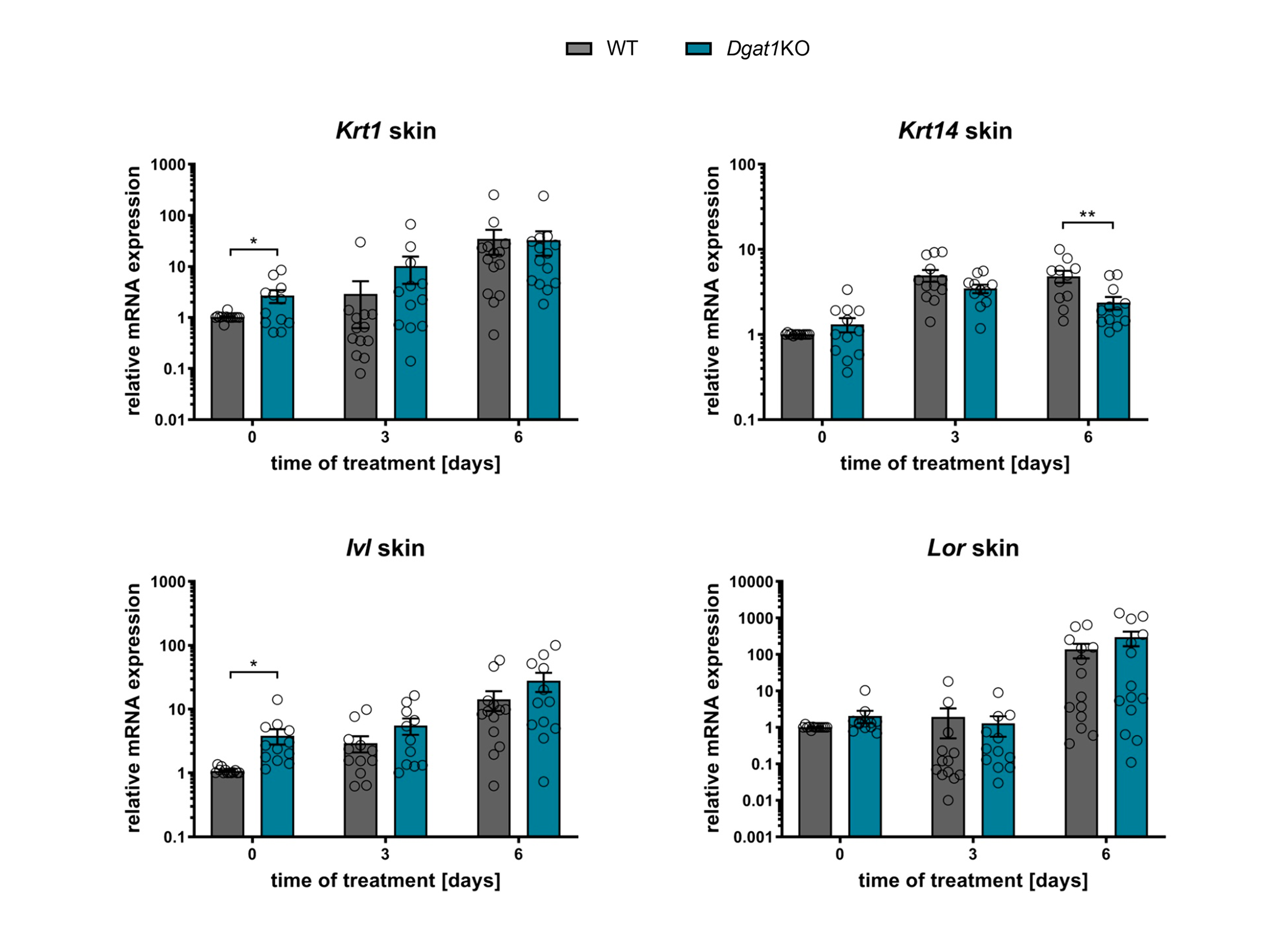

Supplement: Supplementary file 3 — DGAT1 affects keratinocyte differentiation. Relative expression of genes characteristic for keratinocyte proliferation and differentiation in murine skin (Krt1 – keratin 1, Krt14 – keratin 14, Ivl – involucrin, Lor – loricrin). The data are shown as a mean ± SEM; * p < 0.05, ** p < 0.01 by t-test. Gray bars = WT mice; turquoise bars = Dgat1KO mice (PNG 582 KB) [file 13105_2026_1196_Fig7_ESM.png]
